# Mr. Wilkinson, on the Cortex Salicis Latifolia

**Published:** 1804-10-01

**Authors:** G. Wilkinson

**Affiliations:** Sunderland


### *To the Editors of the Medical and Physical Journal.*

GENTLEMEN,

IN your Review of my work on the Cortex Salicis Latifolia, vol. x, p. 374, Medical and Physical Journal, you say, "The virtues of the *various species* of *willow bark* have *long* been *ascertained,* and *fully* established, but the *salix* has not been formally received into our Materia Medica."

These remarks, indeed, are the more extraordinary, as neither Mr. Stone, Mr. James, Mr. White, nor myself, have been aware, how, or by whom, we have been anticipated in our labours.

The former gentleman, Mr. Stone,* wrote on the *salix alba,* or common white willow, which Mr. James† found, as well as some others of its species, on repeated trials, to be superior to the *salix latif.* and it does not yet appear, that he has been anticipated by any English authors whatever on this subject; although *Classius,* and *Gunzius,* two foreign physicians, are said to have written on the bark of the willow; but not being able to procure their Treatises, Mr. James, and myself, are quite at a loss, what, or how many species of the willow they have described. This leads me to hope, that by your friendly assistance, we may be enabled to ascertain the truth of what you have asserted, viz. "That the virtues of the *various species* of *willow bark* have *long* been *ascertained,* and fully *established.*

I have remarked in my work,‡ that Evelyn, in his Discourse

---

* Vide Philosophical Transactions, vol. liii. p. 197.

† Observations on the Bark of a particular Species of Willow, by S. James.

‡ Experiments and Observations on the Cortex Salicis Latifolia, p. 24, et seq.

course on Forest Trees, has named thirty-one varieties of the *salix,* though he has described but fifteen; Steph. Robson in his British Flora, eighteen; and Withering in the last edition of his Arrangement of British Plants,* published by his son, has noticed and described twenty-two species; hence there remains nine more, to make up the number of thirty-one varieties of this *genus;* but how, or by what authors the *virtues,* even of the twenty-two *varieties,* have been *ascertained,* and *fully established,* with me is a doubt, that you will I trust admit, requires explanation.

It is farther asserted by you, in your *Critique,* that you have no doubt, "That the willow bark (by which I suppose you mean the *salix latifolia* in general) properly administered, will cure intermittents, will have a salutary effect in supporting the strength of the constitution under copious suppurations, and other debilitating circumstances, &c." Nevertheless, you question the superiority I apprehend it possesses over the *cinchona*; which difference of opinion can only be thus accounted for. I have considered it (the salix) in a general point of view, and you in the abstract, by your supposing the *cinchona* to be always *genuine*; which is not the case. My reasons for preferring the *salix* to the *cinchona* are, that in *decoction* it is equal, if not superior to the latter, even when *genuine,* and given in *powder*; as it is well known, that in this form it is not only apt to sit uneasy on the stomach, often producing nausea and vomiting, but when retained, brings on troublesome diarrhœa, whereby its good effects become abortive; whereas the former, by proving much more agreeable in a liquid form, *rarely,* if *ever,* disagrees with the patient, and is by no means apt to run off by the intestines,

Whether the Cinchona administered to the patients who were the subjects of my Cases, might or might not be "*genuine or good,*" is not for either of us to determine; more especially, as it was employed by other practitioners prior to their entering on the use of *my salix decoction,* (in powder). Though this latter mode of using it is generally deemed more efficacious than any other, yet the *salix* was universally preferred, for its being more agreeable to their tastes, and by sitting more easy on the stomach; which, in the form of decoction, must be allowed to be of no small advantage; for when we reflect on the already named in-

 conveniencies

* Arrangement of British Plants, vol. ii. p. 44, et seq.

conveniencies attendant on the use of the powder, even of *genuine and good* cinchona, when obliged to be employed in large and repeated doses, in order to produce decisive effects in formidable diseases, inconveniencies which do not attach to the use of the salix; it will certainly appear to be of no small importance to it, as a medicine.

If I have appeared to undervalue the *bitterness* contained in the different species of the cinchona, which I am not conscious of having done, it is on the following principles, viz. That vegetables possessing *bitterness* are accounted *tonic,* and even have been considered as *antiseptics,* though not possessing *tan;* the latter principle having been proved by my experiments* to resist putrefaction of animal substances in preference to *bitterness.* The same terms have been applied to such vegetables as contain *tan,* and bitterness,† though with more propriety than in the former case. Be this as it may, it seems pretty generally admitted, that vegetables not possessing tan have fallen short of the effects of those which contained it; e. g. cham. absinthium gentian cort. aurant. amar. angustura, quassia, &c. are inferior to the cinchona, as *febrifuges,* while the *salix latifolia,* and some others of a similar quality, appear even *sine amaritiè,* to excel the cinchona.

How far bitterness may be found *essentially* necessary towards the perfection of vegetable tonics, as *febrifuges,* is not for me to determine. In the cases of intermittents and typhus, in which I have administered the *salix,* it did not appear defective from a want of this property; nor, for any thing we know, is it *essential* to its effects in preventing the return of the paroxysms of febrile diseases, whatever it may be in some diseases of the chylopoetic viscera, in which bitters combined with *tonics* are known to prove salutary. Nor would it be fair to determine on its effects *comparatively* with the *cinchona,* by combining it with *bitterness,* or any other principle that might influence its effects as a medicine, more especially where the cinchona is trusted to as the sheet anchor.‡

All

---

* Vide my Experiments and Observations on the Salix Latifolia.

† Intense bitterness in vegetables possessing *tan,* is not perhaps so common as in those that contain little or none. The *salix pentandria,* or bay leaved willow bark, is however found to be very similar to the cort. flav. as it possesses *tan,* and bitterness nearly equal to it.

‡ This is to be understood in cases of intermittents, fevers, gangrene, weakening discharges from abscesses, &c. and even in those fevers termed putrid or malignant.

All *formulæ* of the Peruvian bark, such as decoction, tincture, extract, powder, *&c.* notwithstanding the experience of their inefficacy, when given *singly*, in some diseases, have been found, when combined with intense bitters, chalybeates, and other powerful auxillaries, to be attended with uncommon success. This has induced me to suspect its *real* utility in such cases, more especially as it cannot be denied but that the same ingredients, *sine cortice*, have been known to produce similar effects.

I advance not these remarks from caprice, or prejudice against the cinchona, *(or its admirers)*; they are adduced as, at least, presumptive proofs of imperfections that exist in no small degree in medical practice, and which regular practitioners would do well to turn over in their minds, thereby to enable them to ascertain, with accuracy, the specific or active properties resident in powerful remedies.

Thus much have I deemed it necessary to say, in support of my opinion of, and predilection for, the *Salix Latifolia*, which I would have done long ago, had health permitted. I now, Gentlemen, take the liberty of thus addressing you on the subject, hoping you will allow these my observations a place in your valuable Repository; and respectfully solicit your attention, particularly to what relates to the commencement of my Paper, as it is with me a matter of no small importance.

I am, &c.

*Sunderland, Sept.* 13, 1804. G. WILKINSON.

